# Automatic detection of abnormalities in mammograms

**DOI:** 10.1186/s12880-015-0094-8

**Published:** 2015-11-06

**Authors:** Zobia Suhail, Mansoor Sarwar, Kashif Murtaza

**Affiliations:** Punjab University College of Information Technology (PUCIT), University of the Punjab, Lahore, Pakistan

## Abstract

**Background:**

In recent years, an increased interest has been seen in the area of medical image processing and, as a consequence, Computer Aided Diagnostic (CAD) systems. The basic purpose of CAD systems is to assist doctors in the process of diagnosis. CAD systems, however, are quite expensive, especially, in most of the developing countries. Our focus is on developing a low-cost CAD system. Today, most of the CAD systems regarding mammogram classification target automatic detection of calcification and abnormal mass. Calcification normally indicates an early symptom of breast cancer if it appears as a small size bright spot in a mammogram image.

**Methods:**

Based on the observation that calcification appears as small bright spots on a mammogram image, we propose a new scale-specific blob detection technique in which the scale is selected through supervised learning. By computing energy for each pixel at two different scales, a new feature “Ratio Energy” is introduced for efficient blob detection. Due to the imposed simplicity of the feature and post processing, the running time of our algorithm is linear with respect to image size.

**Results:**

Two major types of calcification, microcalcification and macrocalcification have been identified and highlighted by drawing a circular boundary outside the area that contains calcification. Results are quite visible and satisfactory, and the radiologists can easily view results through the final detected boundary.

**Conclusions:**

CAD systems are designed to help radiologists in verifying their diagnostics. A new way of identifying calcification is proposed based on the property that microcalcification is small in size and appears in clusters. Results are quite visible and encouraging, and can assist radiologists in early detection of breast cancer.

## Background

Breast cancer is one of the major causes of death among women all over the world. The real cause of breast cancer is still unknown. Thus, early detection of breast cancer and its treatment is the only way to possibly longer life and improved quality of life of patients. CAD systems help greatly in diagnosing breast cancer. In addition, these systems may also be used as a second opinion by radiologists for the verification of diagnostic results. In such CAD systems, accuracy of results is of primary importance. A minor wrong detection or false miss can lead to wrong or poor treatment. Due to the sensitivity of the problem, many researchers are doing work in the field of *mammogram segmentation* and competing with each other to achieve better results.

Our research mainly focuses on low-cost processing mammogram images [1–5] that results in the segmentation of both abnormal mass and calcification. Statistics show that 30–50 % of cancer has microcalcification and an abnromal mass is also a clear symptom of breast cancer. Thus, early detection of such abnormal mass and microcalcification can help radiologists in better diagnostics, resulting in proper and timely treatment of patients. Segmentation of mammograms for identifying calcification and other masses is an active area of research [6–9].

I. N. Bankman and T. Nizialek [6] present a new algorithm that is independent of the parametric measurement of the targeted data. They use multitolerance region growing and active contour model for segmentation of mammograms. They does not use any manual threshold or window selection for the detection process. Unfortunately, their approach for segmentation required much time. In some cases, their algorithm reported somewhat larger area compared to the ground truth. Another region growing method is proposed by S. A. Hojjatoleslami and J. Kittler [10]. They use average and peripheral contrast to control the growing process. The major feature of their approach is that at each step only one pixel exhibits the properties to join the region. Unlike the threshold based segmentation, this approach does not necessarily include all the pixels with the same gray level in the region.

AP. Stefanoyiannis et al. [11] propose a method to improve breast periphery in mammogram images by using wavelet-based fusion techniques. This technique results in mammogram images with improved contrast. They use both thresholding and wavelet-based fusion to overcome the overexposure problem of breast periphery during mammography imaging. A. Papadopoulosa [12] assessed the effect of image enhancement and parameters tuning for microcalcification detection. They compared five major image enhancement techniques for CAD assessment, including Contrast-Limited Adaptive Histogram Equalization (CLAHE) and the Local Range Modification (LRM). They report the results of each technique with respect to microcalcification detection and conclude that the LRM algorithm, in most cases, exhibits the best performance but with the selection of appropriate values of the tuning parameters.

I.K Maitra et al. [13] propose Binary Homogenity Enhancement Alogrithm (BHEA), followed by Seeded Region Growing Algorithm (SRGA) to differentiate between normal and abnormal tissues. Their algorithm proceeds to region growing after differentiating between different regions of breast by applying their proposed Anatomical Segmentation of Breast (ASB) algorithm.

R. Lakshmanan [14] proposes an approach to enhance microcalcification features using zero-crossings of contourlet transform, morphology, and artificial neural network that result in an enhanced image as well as preserves features. They tested their scheme using images from the mini-MIAS database.

Finding object boundaries in noisy images is a difficult task and is an important initial step for many segmentation algorithms. K. Somkantha [15] proposes an algorithm for boundary detection in medical images using edge detection techniques.

A. Wroblewska et al. [9] propose a technique for microcalcification detection based on supervised learning. First, they segment objects within a mammogram using the top-hat and thresholding techniques and then use the selected features of the segmented objects as input to a neural network.

Another technique used in mammogram classification is Wavelet Decomposition. T. C. Wang et al. [8] propose an algorithm for microcalcification detection by decomposing an image into different frequency subbands using wavelet decomposition and then reconstructing the image by taking maximum contribution from the high frequency subbands. In wavelet decomposition, the image is decomposed into different frequency subbands, where the region having microcalcification exhibits relatively high frequency as compared to other areas.

E. Hashemi et al. [16] also use wavelet based microcalcification detection. They show that wavelet features of the image have the property that the reconstruction of the image from major wavelet features have common values of skewness and kurtosis on the intersection of their rows and columns. Therefore, they report the intersection area of significant rows and columns (i.e., rows and columns having the highest values of skewness and kurtosis) to be the strong candidate for microcalcification clusters.

It is observed that calcifications, specifically microcalcification areas, occur as tiny blobs in mammograms. Several approaches exist for blob detection. Some of them are scale invariant. For example, Lindeberge [17] describes a blob detection technique at different scales.

In our paper, we propose a method for calcification detection in general and not specific to mammograms. Because the sizes of microcalcification blobs in mammograms are roughly the same, therefore, detection of blobs in mammograms using scale space theory is an overkill. We propose a simple and efficient method for blob detection at a particular scale guided by mammogram imaging.

## Methods

*Ethics Statement:“This research was approved by the Ethics committee of INMOL hospital [Lahore, Pakistan] before conducting the actual research. Dr. Zeeshan Rashid Mirza was our focal person in the hospital. He provided us all the information regarding the data formats and properties of certain abnormalities.”*

**Fig. 1:**
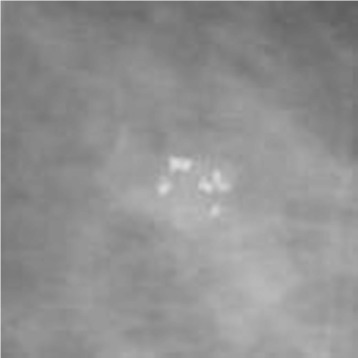
Cancerous Image: Image Id: 1004217-cut-806929 from IRMA version of DDSM

**Fig. 2:**
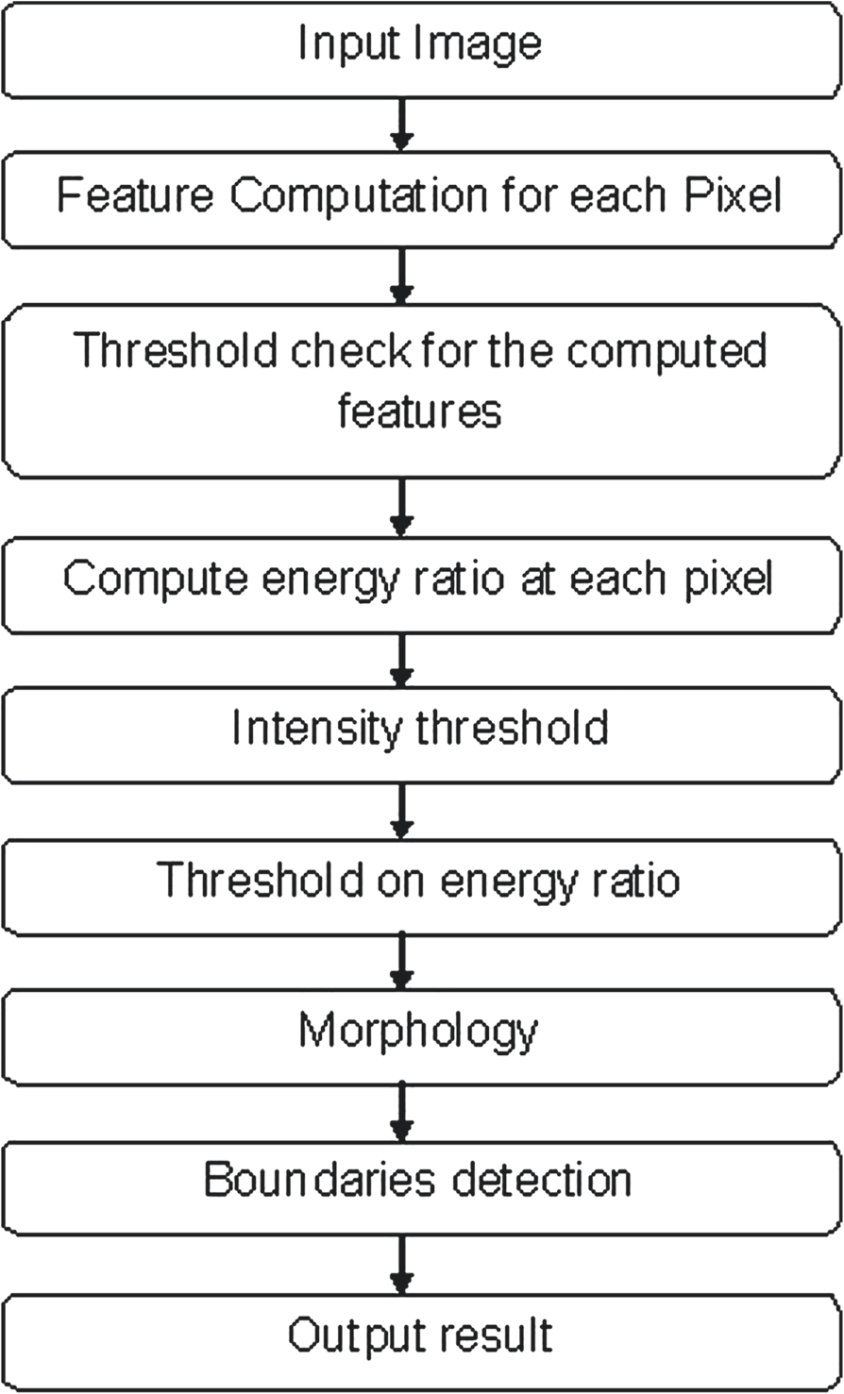
Steps of the proposed algorithm

It has been observed that an abnormality, specifically microcalcification area, occurs as a tiny blob in mammograms having more brightness, hence intensity, compared to its neighboring pixels [Fig. 1]. We compute energy at each pixel in a mammogram for two different window functions. By taking the ratio of energy computed for a small window (3 × 3) to the energy computed for a large window (11 × 11), we detect the suspicious area containing an abnormality by thresholding the energy ratio to 80 % of maximum energy ratio and by applying intensity threshold steps afterwards. Then, we apply postprocessing on final results using morphological operations. We apply pre-processing steps at the start of the algorithm to filter out normal images, i.e, images having no abnormality. The purpose of applying this filtering is to eliminate the extra processing involved in processing normal images. A block diagram describing the method used is shown in Fig. 2.

### Preprocessing

The purpose of the preprocessing step is to filter out normal images from the dataset. This saves time from further processing the normal images and to minimize the false positive results. So, if an image is normal, it is reported immediately. To filter out normal images, we compute kurtosis and skewness for a 20×20 sliding window. The specific pixel is reported as normal if kurtosis value of a pixel centered at the 20 × 20 window exceeds 14 and skewness value exceeds 2.3. If we do not find any pixel in the whole image that belongs to these threshold limits of skewness and kurtosis, we consider the image to be a normal image, having no abnormalities.

### Energy computation

After the preprocessing step, our algorithm finds the suspicious regions within the mammogram. For our algorithm, we choose the small window of size 3 × 3 and the larger window of size 11 × 11. We took these window sizes after cross validating results on different window sizes. The energy at a pixel is computed as the sum of the intensities of the pixels covered by the window centered at that pixel. Suppose *P*(*x,y*) represents the intensity of a pixel positioned at coordinate x,y in an image. Then the 3 × 3 and 11 × 11 windows centered at *P*(*x,y*) are represented by Tables 1 and 2, respectively.

**Table 1:** Small window (3 × 3)

*P*(*x*−1,*y*−1)	*P*(*x*−1,*y*)	*P*(*x*−1,*y*+1)
*P*(*x,y*−1)	*P*(*x,y*)	*P*(*x,y*+1)
*P*(*x*+1,*y*−1)	*P*(*x*+1,*y*)	*P*(*x*+1,*y*+1)

**Table 2:** Large window (11 × 11)

*P*(*x*−5,*y*−5)	..	..	*P*(*x*−5,*y*−1)	*P*(*x*−5,*y*)	*P*(*x*−5,*y*+1)	..	..	*P*(*x*−5,*y*+5)
*P*(*x*−2,*y*−5)	..	..	*P*(*x*−2,*y*−1)	*P*(*x*−2,*y*)	*P*(*x*−2,*y*+1)	..	..	*P*(*x*−2,*y*+5)
..	..	..	..	..	..	..	..	..
*P*(*x*−1,*y*−5)	..	..	*P*(*x*−1,*y*−1)	*P*(*x*−1,*y*)	*P*(*x*−1,*y*+1)	..	..	*P*(*x*−1,*y*+5)
*P*(*x,y*−5)	..	..	*P*(*x,y*−1)	*P*(*x,y*)	*P*(*x,y*+1)	..	..	*P*(*x,y*+5)
*P*(*x*+1,*y*−5)	..	..	*P*(*x*+1,*y*−1)	*P*(*x*+1,*y*)	*P*(*x*+1,*y*+1)	..	..	*P*(*x*+1,*y*+5)
*P*(*x*+2,*y*−5)	..	..	*P*(*x*+2,*y*−1)	*P*(*x*+2,*y*)	*P*(*x*+2,*y*+1)	..	..	*P*(*x*+2,*y*+5)
..	..	..	..	..	..	..	..	..
*P*(*x*+5,*y*−5)	..	..	*P*(*x*+5,*y*−1)	*P*(*x*+5,*y*)	*P*(*x*+5,*y*+1)	..	..	*P*(*x*+5,*y*+5)

The energy at *P*(*x,y*) for a window *w*_(*wr,wc*)_, $\sum _{w}$, is computed as follows:

$$\sum_{w_{(x,y)}} = \sum_{i=-wr}^{wr} \sum_{j=-wc}^{wc} P(x+i,y+j).$$

For the small window w =s and wr =*wc*=3. For the large window w =l and wr =*wc*=11. The Ratio Energy (RE) is computed by Eq. (1).

(1) $$\begin{array}{*{20}l} RE_{(x,y)}&= \frac {\sum_{s_{(x,y)}}} {\sum_{l_{(x,y)}}} \times 100 \end{array} $$

**Fig. 3:**
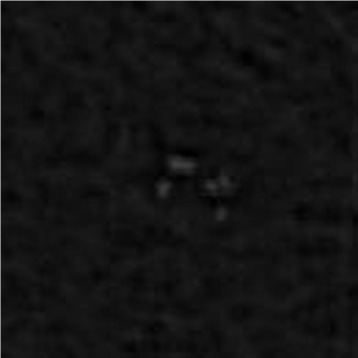
Image showing complement of Ratio Energy computed at each pixel

RE is computed for every pixel in the image. We then eliminate outliers from the image for stable estimation of further thresholds. Figure 3 shows the result after taking complement of Ratio Energy computed at each pixel.

### Threshold computation

To avoid inclusion of outlier pixels, we apply threshold on the pixel intensities. We do this so the pixels that are of no interest for the detection of abnormal masses may not contribute to energy threshold. So, in the initial step of thresholding we ignore the RE’s of those pixels that lie below our defined intensity threshold *χ*, as represented by Eq. (2).

(2) $$\begin{array}{*{20}l} {\chi}= 90 /100 \times \max(Intensity_{p(x,y)}) \end{array} $$

Maximum intensity used in Eq. (2) is taken from a function that takes top two maximum intensities from the image. It then compares the difference between these intensities. If the difference is greater than a certain threshold, it means there exists an outlier in the image and the intensity selection function simply selects the second intensity to be the intensity threshold. Otherwise, it returns the first energy to be the intensity threshold.

After applying intensity threshold, we compute *ε*, the maximum of all energy ratios, according to Eq. (3).

(3) $$\begin{array}{*{20}l} {\epsilon} = \max (RE_{p(x,y)}) \end{array} $$

Next, we compare the RE of each pixel with the energy threshold *δ*, defined in Eq. 4.

(4) $$\begin{array}{*{20}l} {\delta}= 80/100 \times \epsilon \end{array} $$

**Fig. 4:**
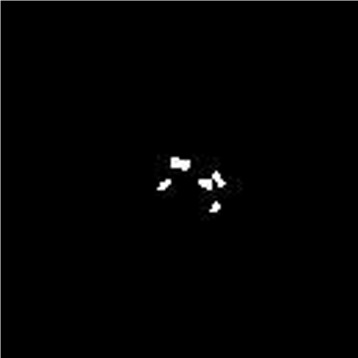
Image after thresholding ratio energy image to maximum energy and intensity

If RE of a pixel, *P*(*x,y*), is greater than or equal to ***δ***, then the pixel is labeled as a foreground pixel, i.e., it belongs to microcalcification or abnormal mass. Figure 4 shows the abnormal area after energy and intensity thresholds. We adjust our thresholds in a way to minimize the possibility of high false negative rates. However, this biasness in thresholding introduces some false positives in our results, for which we apply the post processing step.

### Post processing

In this step, we reduce the number of false positives. We use the morphological operations with the diamond shaped structuring element for this purpose. Because microcalcifications appear as bright and tiny spots and normally have a size no more then 20 pixels on the mammograms. Depending on this property we choose the size of structuring element larger than 20 pixels.

Suppose *P*(*x,y*) represents a gray scale mammogram image and S is the structuring element, then the basic morphological operations Erosion, ⊖, and Dilation, ⊕, are defined as follows:

**Erosion:** [P⊖S]_(*x,y*)_=min_(*u,v*)∈*S*_*P*(*x*+*u,y*+*v*)

**Dilation:** [P⊕S]_(*x,y*)_=max_(*u,v*)∈*S*_*P*(*x*−*u,y*−*v*)

Based upon these basic morphological operations Opening morphological operation, *o*, is defines as Erosion followed by Dilation P *o* S=(P⊖S) ⊕ S.

**Fig. 5:**
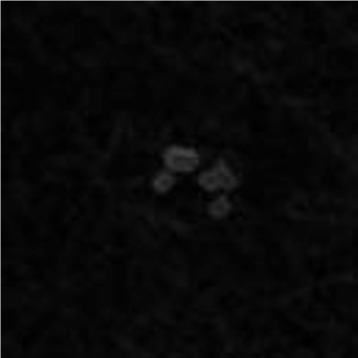
Results of tophat morphological operation on original image

We apply the TopHat morphological operation on the gray scale mammogram image by computing morphological opening of the image and subtracting it from our original image, f, as shown in Eq. (5) [Fig. 5].

(5) $$\begin{array}{*{20}l} TopHat(P) = P - (P \textit{o} S) \end{array} $$

**Fig. 6:**
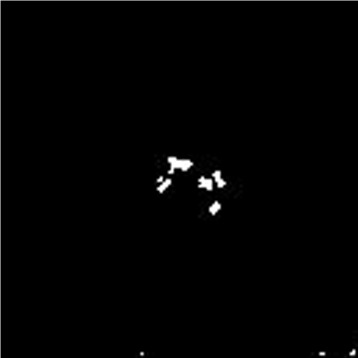
Image shows thresholding after the tophat operation

We use the *imtopHat* function of Matlab to apply the morphological TopHat operation. Then we binarize the image that is obtained by Eq. (5) by taking pixels having intensity > 4.0 *σ*, where *σ* is standard deviation [Fig. 6].

After this process, we have two images:

- I_*e*_= Image after computing and thresholding the energy function, and
- I_*t*_= Image after applying TopHat and thresholding.

**Fig. 7:**
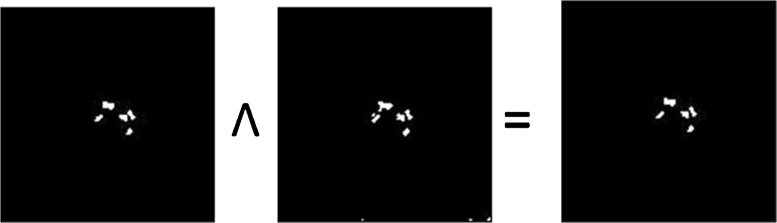
The image after applying the logical AND operation on the RE image and the tophat image

The intersection of these images, I_*r*_, shown in Eq. (6) is the expected outcome of calcification [Fig. 7], where calcification includes both microcalcification and macrocalcification.

(6) $$\begin{array}{*{20}l} I_{r} = I_{e} \wedge I_{t} \end{array} $$

where ∧ is the logical AND operation.

**Fig. 8:**
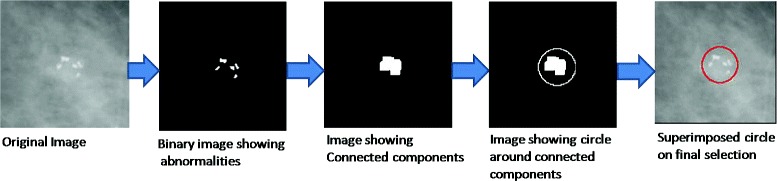
Steps to draw boundary around the final detection

Finally, the boundary circle is drawn around an abnormality by estimating the center and radius of the circle with respect to the density of the foreground pixels. Figure 8 shows certain steps involved in identifying and drawing circle around the abnormal region.

## Results and discussion

The decision of a CAD system can fall into one of the four categories. An image region can be called abnormal (positive) or normal (negative), and a decision can either be correct (true) or incorrect (false). CAD can generate two types of erroneous outputs, i.e., False Positive (FP) and False Negative (FN). True Positive (TP) and True Negative (TN) are the two correct decisions. Two performance measures of a detection system that are related to the decisions identified above are ‘Sensitivity’ and ‘Specificity’. Sensitivity (or Recall) is the probability of a positive test, given that the patient is ill while specificity is the probability of a negative test that the patient is well. High values of sensitivity and specificity are desirable. ‘Accuracy’ and ‘Precision’ are also used for performance evaluation of CAD systems.

To assess the performance of our algorithm, we tested it on 84 images from the DDSM database. 54 (64 %) of these images were normal and 30 (36 %) were cancerous. The analyses of our results are shown in Table 3.

**Table 3:** Analysis of results

Performance measure	Abnormal cases		Normal cases	
	30 (36 %)		54 (64 %)	
	TP	FN	TN	FP
	29 (97 %)	01 (3 %)	49 (91 %)	05 (9 %)
Sensitivity	91 %			
Specificity	97 %			
Precision	85 %			
Accuracy	93 %			

## Detection criteria

Evaluating the performance of a CAD scheme developed for mammogram classification requires certain criteria for determining TP and FP cluster detection. For the evaluation of our results, genuine clusters of calcification were identified by an expert radiologist. The criteria that we used for counting the number of TP detections is to regard a cluster as correctly detected if three or more pixels are found within the region marked as containing calcification by a radiologist. All other regions, if detected, are considered to be FP.

As shown in Table 3, our algorithm has 91 % sensitivity, 97 % specificity, 93 % accuracy, and 85 % precision. Two expert radiologists^1^ found our results to be very satisfactory and reliable.

## Algorithm complexity

For each pixel in the image, the RE calculation takes *O*(window size) time because we detect blobs at a specific scale regardless of the size of the image, i.e., the window size is fixed (constant). Therefore, time complexity of RE computation at each pixel is *O*(1), which accumulates to *O*(n) for the image having n pixels. The cost for thresholding for both energy and intensity in also linear in terms of the number of pixels in the image. Therefore, the time complexity of thresholding is *O*(n).

The refinement of final results includes the morphological operation, as morphology requires convolution of the structuring element over the whole image. Because of fixing the scale, the size of structuring element is also fixed resulting in the time complexity of the total convolution to be *O*(n). The growth rate of the whole algorithm, T(n), is T(n) = time cost for computing R.E at each pixel + time cost for thresholding + Time cost for morphology =*O*(n)+*O*(n)+*O*(n)=*O*(n).

Therefore, the algorithm is linear in terms of number of the pixels in the image.

## Conclusions

CAD systems can help greatly in the early detection of illness. Mammogram classification is one of the major applications where CAD systems are being used. A mammogram image is usually quite noisy and it is not easy to detect region of interest (ROI) from it. Even an expert radiologist cannot identify with 100 % certainity that the area of concern is always identified correctly. According to a survey, almost 25 % of microcalcification are missed by radiologists at early stages. A large number of breast image examinations is one of the reasons of this miss ratio.

CAD systems are superior in that once developed, they can operate with the same accuracy on any number of images. Hardware failures may be a reason for the system to crash, but increased examination load does not affect the performance of the system.

CAD systems developed for mammogram classification help radiologists to get a second opinion and decrease a radiologist’s miss ratio. Radiologists compare their opinions with the results of a CAD system and base their final diagnoses on a “double reading” of results. The ultimate goal of a CAD system for mammography is to detect cancer when it is too small to be felt by a physician radiologist. This early detection greatly improves a women’s chances of successful treatment of her breast cancer.

Our research mainly focuses on the very basic property of microcalcification that these are brighter spots on a mammogram image as compared to the remaining breast periphery. We use ratio energy (RE) as a feature that discriminates the area containing abnormality from the rest of the area in a mammogram image. After obtaining maximum RE we then compare the energy of each pixel to thresholded maximum RE in order to judge whether the pixel belongs to calcification or not. Finally, we clean up our results using morphology. We also observe some region based properties of normal images (without cancer) that are different from abnormal images (with cancer) and use these properties to filter out normal images at early stages of our algorithm and avoid their further processing. This step reduces the number of FP results.

Using a very simple feature of an image, our system is highly efficient and makes a constant number of scans of the image to produce final results. Further, the results are region based rather then pixel based because microcalcification occurs as dense clusters.

According to two expert radiologists, the results produced by our system are quite satisfactory and reliable, and can assist radiologists in early diagnosis of breast cancer.

## Endnote

^1^ Dr. Zeeshan Rashid Mirza from Institute of Nuclear Medicine and Oncology (INMOL) and Dr. Zia Faruqui from Diagnostic Center, Lahore, Pakistan.

## Abbreviations

CAD: Computer aided diagnostics
ROI: Region of interest
ROC: Receiver operating characteristics
DDSM: Digital database for screening mammography
MIAS: Mammographic image analysis society
DICOM: Digital imaging and communications in medicine
CT: Computed tomography
MBI: Molecular breast imaging
RE: Ratio energy
KPV: Peak kilovoltage
MCC: Microcalcification cluster
JPEG: Joint photographic expert group
TIFF: Tagged image file format
GIF: Graphics interchanged format
PNG: Portable network graphics
IRMA: Image retrieval in medical applications
